# *NLRP7* and the Genetics of Hydatidiform Moles: Recent Advances and New Challenges

**DOI:** 10.3389/fimmu.2013.00242

**Published:** 2013-08-20

**Authors:** Rima Slim, Evan P. Wallace

**Affiliations:** ^1^Department of Human Genetics, McGill University Health Centre, Montreal, QC, Canada; ^2^Department of Obstetrics and Gynecology, McGill University Health Centre, Montreal, QC, Canada

**Keywords:** *NLRP7*, hydatidiform mole, spontaneous abortions, reproductive loss, maternal-effect genes

## Abstract

NOD-like receptor proteins (*NLRPs*) are emerging key players in several inflammatory pathways in Mammals. The first identified gene coding for a protein from this family is *Nlrp5* and was originally called *Mater* for “Maternal Antigen That Mouse Embryos Require” for normal development beyond the two-cell stage. This important discovery was followed by the identification of other *NLRP*s playing roles in inflammatory disorders and of the first maternal-effect gene in humans, *NLRP7*, which is responsible for an aberrant form of human pregnancy called hydatidiform mole (HM). In this review, we recapitulate the various aspects of the pathology of HM, highlight recent advances regarding *NLRP7* and its role in HM and related forms of reproductive losses, and expand our discussion to other *NLRP*s with a special emphasis on those with known roles in mammalian reproduction. Our aim is to facilitate the genetic complexity of recurrent fetal loss in humans and encourage interdisciplinary collaborations in the fields of *NLRP*s and reproductive loss.

## Historical View about HM

Hydatidiform mole (HM) is an aberrant human pregnancy with no embryo that has fascinated and puzzled scientists in all civilizations. The recognition and description of this condition is very ancient and appears in Hippocrates’ manuals under the name “dropsy of the uterus” (1). In addition, a famous physician in the Byzantine period, Aetius of Amida, who was the private physician of Emperor Julian, wrote about moles and interestingly used the term inflammation to describe them, “an inflammation or strenuous walking” (2, 3). The etiology of HM continues to fascinate scientists in several aspects. HM is the only disease or tumor that may be formed by androgenetic, non-self-cells from a woman’s sexual partner as opposed to all other tumors and cells in our body. Despite their common histopathological features, different HM tissues may have different parental contributions. Depending on its mode of formation, a HM’s genotype might be diploid biparental, diploid androgenetic monospermic, diploid androgenetic dispermic, triploid dispermic, triploid digynic, tetraploid, aneuploid, or mosaic. These diverse possibilities generate an important diagnostic complexity and therefore continue to challenge scientists and clinicians in various disciplines. In this chapter, we review the pathology of HMs and describe recent advances in our understanding of its pathogenesis.

## Epidemiology of HM

The common form of HM is sporadic and not recurrent. The geographical distribution of its incidences varies widely, with a frequency of 1 in every 600 pregnancies in western countries (4) and 2–10 times higher frequencies in developing and undeveloped countries (5–7). Depending on populations and studies, 1–6% of women with a prior HM will develop a second HM (8–14). Cases in which a single family member has recurrent HM (RHM) are called singleton cases and those in which at least two women have one or several HM are called familial cases. Familial RHM is rarer and its exact frequency is not known.

## Clinical and Ultrasound Manifestations

**Figure 1:**
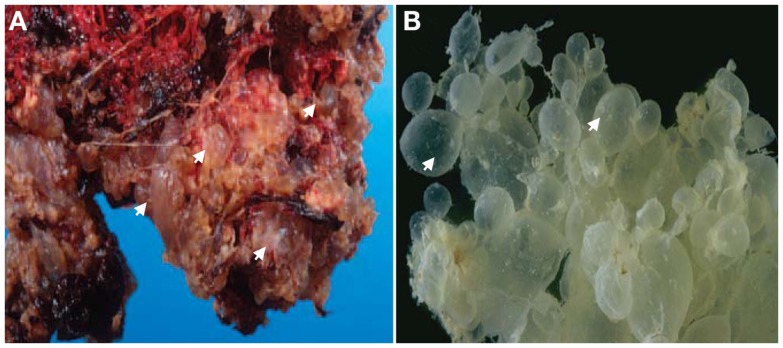
**Gross-morphology of HMs**. **(A)** A photograph of gross-morphology of a HM directly after surgical evacuation. Note the presence of vesicles (only four are indicated by arrows) which represent edematic chorionic villi (CV) that have accumulated fluid and are covered with blood. **(B)** Another gross-morphology photograph of a HM after removing the blood. Note the hydropic degeneration of the CV and their appearance as a grape-like structure (only two are indicated by arrows). In **(B)** photo courtesy of Professor Edward C. Klatt, School of Medicine, Mercer University.

The clinical manifestation of moles has changed with the advances of medicine, largely because of the introduction of ultrasonography in the second half of the twentieth century as a routine exam to monitor all pregnancies starting from the eighth week of gestation. Consequently, many moles are now detected by ultrasound examination at the first gynecological visit or even earlier in cases of vaginal bleeding, which is the most common presenting symptom that would precipitate early medical consultation and diagnosis. Ultrasound indications of moles include the presence of echogenic structures in the placenta, the absence of a gestational sac, and/or the absence of fetal heart activity. These initial ultrasound observations are followed by a blood test of the human chorionic gonadotropin (hCG), the pregnancy hormone that is secreted mainly by syncytiotrophoblast cells of the chorionic villi (CV) into the intervillous space, whereupon it is carried to the maternal systemic blood circulation. HCG is much higher in women with molar pregnancies than in women with normal pregnancies of matching gestational stages, which is believed to be the consequence of the increased proliferation of syncytiotrophoblast cells. Depending on ultrasound findings, the gestational stage of the pregnancy, and the level of blood hCG, further ultrasound examinations and hCG follow-up tests may be required before a clinical decision is reached regarding the arrest of the pregnancy and the requirement of a surgical evacuation of the product of conception (POC). A non-viable pregnancy accompanied with a high hCG test will necessitate dilatation and curettage suction of the POC. The evacuated tissues (Figure 1) are submitted for histopathological examinations and the diagnosis is made based on histopathological findings and criteria.

## Histopathology and Diagnosis

**Figure 2:**
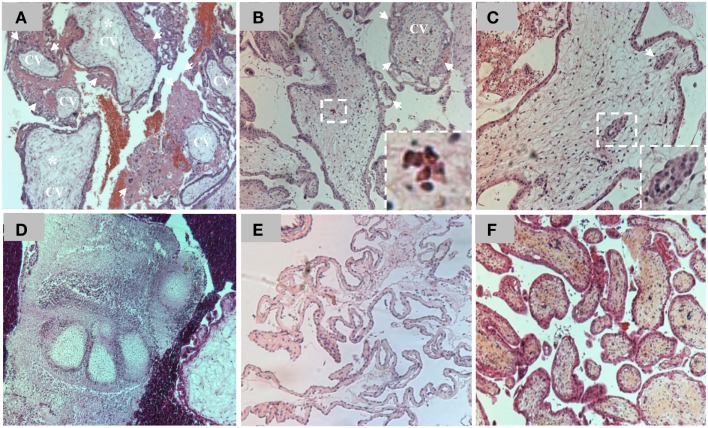
**(A)** Hematoxilin and eosin (H&E) staining of a section of chorionic villi (CV) from a CHM. Note the presence of excessive circumferential trophoblastic proliferation around all CV (arrows) and the beginning of hydropic degeneration in two CV (asterisks). **(B)** H&E staining of a section of CV from a PHM. Note the presence of circumferential trophoblastic proliferation (arrows) around one chorionic villus (indicated by CV) while the others have no or few sprouts of trophoblastic proliferation (arrows). Note the presence of nucleated red blood cells inside the chorionic villus (on the right corner) in the conception that led to PHM. **(C)** One CV from a PHM displaying trophoblastic inclusions (arrows and magnified view on the right corner). **(D)** A view from a PHM showing phalanges of fetal foot. **(E)** Another view from a PHM showing fetal membranes. **(F)** H&E staining of a section of CV from a spontaneous abortion. Note the absence of trophoblastic proliferation around the CV.

The original definition of HM was a pregnancy devoid of a fetus in which the chorion is replaced by grape-like vesicles. A long time ago, the severe form of this condition was believed to originate from pathologic ovaries (15) and was originally called “true moles” or “classical moles,” which correspond to what we now call complete HMs. This classification evolved and other terms emerged later to describe milder forms of the same condition such as “transitional,” “partial,” and “incomplete” moles (16–18). The current histopathological classification of spontaneously arrested pregnancies includes three entities designated complete HM (CHM), partial HM (PHM), and non-molar spontaneous abortion (SA) (19). CHMs display circumferential trophoblastic proliferation affecting most CV (Figure 2) and do not contain extra-embryonic membranes (chorion and amnion), a fetal cord, fetal nucleated red blood cells, or any other embryonic tissue of inner cell mass origin. Both SAs and PHMs may contain extra-embryonic membranes (chorion and/or amnion), a fetal cord, fetal nucleated red blood cells, other embryonic tissues (cartilage, bones, etc.), or even a normal or an abnormal complete fetus (Figure 2). PHMs display mild and focal trophoblastic proliferation that can be observed on some CV and in several microscopic fields, whereas SAs do not display abnormal circumferential trophoblastic to warrant close hCG follow-up (Figure 2). The histopathological subdivision of spontaneously arrested pregnancies into CHMs, PHMs, and SAs has always been challenging and several scientists have noted the continuous variation in the molar degeneration (18). This challenge is more problematic nowadays because of the early evacuation of arrested pregnancies based on ultrasonography and before the manifestations of all their histopathological features. Consequently, there is a wide inter-observer and intra-observer variability in distinguishing non-molar SAs from PHMs and in distinguishing PHMs from CHMs (20–22). Practically, the difficulty for the pathologists is to know where to draw the lines of separation between the three entities due to the continuous spectrum of abnormalities and due to the fact that histopathology is a qualitative descriptive science (mild, excessive, focal, occasional, etc.) that lacks quantitative measurements to assess the degree and extent of trophoblastic proliferation.

## Karyotype and Genotype Data

Karyotype and genotype analyses have shown that sporadic moles may have different genotypic types with the majority of CHMs being diploid androgenetic and the majority of PHMs being triploid diandric dispermic. Among androgenetic moles, the majority are monospermic and 10–20% are dispermic (23–27). In a minority of cases, some CHMs have been reported to be diploid biparental (25), triploid diandric dispermic (23), tetraploid triandric (3 paternal and 1 maternal sets of chromosomes) (28) or digynic (29), aneuploid, or mosaic with two cellular populations. Sporadic PHMs are mostly triploid diandric dispermic, but they have also been reported with diploid biparental, triploid digynic (29), triploid monospermic (30), tetraploid triandric (31, 32), or aneuploid genomes. Based on the major categories of complete and partial moles, different hypothetical models at the origin of moles’ formation were proposed and have been accepted by the scientific community over the last 30 years. One of these models postulates that an androgenetic monospermic mole results from the fertilization of an empty oocyte by a haploid sperm that undergoes an endoreduplication of its genome to form the diploid androgenetic monospermic mole. Similarly, androgenetic dispermic moles would result from the fertilization of an empty oocyte by two spermatozoa, while triploid diandric (or dispermic) moles would result from the fertilization of a haploid oocyte by two different haploid spermatozoa. These accepted models were recently challenged by Golubovsky (33) who refutes the existence of empty oocytes at the origin of androgenetic moles. Instead, he proposes that dispermic fertilization followed by complex postzygotic abnormalities and diploidization is at the origin of the various genotypic types of moles as well as mixoploidies, trisomies, and various aneuploidies. These different models and their implications in the genesis of HMs are discussed below.

## Post-Evacuation hCG Surveillance and Malignancies

Molar pregnancies are the most common gestational tumors and are benign in about 80% of cases. In these cases, hCG falls to non-pregnant levels after the surgical evacuation of the molar conception. However, in about 20% of cases in western countries, elevated hCG levels persist for several weeks post-evacuation or rise after falling down, which indicates the retention of some trophoblastic tissues. Such conditions are termed persistent gestational trophoblastic diseases (PGTDs) or gestational trophoblastic neoplasias (GTNs) and may necessitate a second surgical evacuation and/or chemotherapy treatments. GTNs occur most commonly after CHMs (15–29%), less frequently after PHMs (0.5–4%), and rarely after SAs, ectopic pregnancies, or normal pregnancies (34–36). Several classification systems of GTNs have been elaborated over time and are used to help standardize and optimize treatments of these conditions. For good reviews on these topics see (37–39). In recent times, the most commonly used guidelines are those of the World Health Organization (WHO) and the Fédération Internationale de Gynécologie et d’Obstétrique (FIGO).

The most common malignant degeneration of HMs or GTNs are invasive moles and gestational choriocarcinomas (CCs). The diagnosis of invasive moles is based on persistent or rising levels of hCG and histopathological identification of CV within the myometrium (the deep layer of uterine tissues beneath the endometrium), maternal blood vessels, or within extrauterine tissues. Invasive moles affect approximately 20 and 2–4% of patients with CHMs and PHMs, respectively (34).

CCs may occur after any type of pregnancy in the following proportions: 35–60% after CHMs, 0.5–2% after PHMs, 15–20% after SAs, 1–2% after ectopic pregnancies, and 25–42% after normal pregnancies (40, 41). The diagnosis of CC is based on high hCG levels and both clinical and laboratory evidence demonstrating the presence of tumor cells in distant maternal tissues such as the lung, lower genital tract, brain, liver, kidney, gastrointestinal tract, and spleen. A definitive diagnosis of CC is based on histopathological findings demonstrating the presence of cytotrophoblastic and syncythiotrophoblast cells, without organized villous structures in distant maternal tissues (42). CCs are the most aggressive GTNs because of their ability to spread hematogenously. They may be fatal in the absence of appropriate follow-up and management. Again, both invasive HMs and CCs have higher frequencies in both developing and underdeveloped countries than in developed countries (40).

## The Importance of Crossing Our Discipline

Despite the ancient clinical recognition of HMs and the presence of several reports describing cases of recurrent moles (15, 43–46) no attempts were made to identify causative genes for the recurrent form until the report by Seoud et al. (47) that led to the mapping of the first maternal locus to 19q13.4 (48). At that time, only six other familial cases of RHMs had been reported in the English PubMed literature since 1980 (49–52). Consequently, we and others believed that the familial form of moles was extremely rare. However, this was not true and approximately 30 new familial cases have been reported since 1999 (47, 53–69) indicating that familial RHMs are not extremely rare as originally believed, but were probably under-reported. In addition, about 88 singleton cases of RHMs have been described since 1999. The importance of the case reported by Seoud and his collaborators (47) is in the fact that the authors crossed the boundaries of their disciplines, a common practice in many medical specialties, but a rare one in the field of Obstetrics and Gynecology. These authors sought the help of scientists from other disciplines at a time where small nuclear consanguineous families were an opportunity for gene mapping by homozygosity analysis. This original family as well as another (51) led to the mapping of the first maternal-effect locus responsible for recurrent moles to 19q13.4 (48) and opened a new avenue of research aimed at identifying maternal genes causing RHMs and recurrent fetal loss.

## Lessons from Studying Extreme Phenotypes

**Figure 3:**
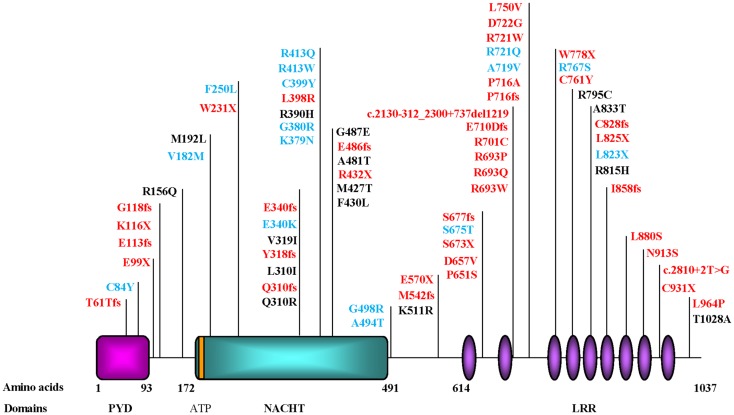
**Schematic representation of NLRP7 protein structure and identified mutations and variants in patients with hydatidiform moles and reproductive loss**. PYD, stands for the pyrin domain, NACHT, stands for found in the NAIP, CIITA, HET-E, and TP1 family proteins; ATP for adenosine 5′-triphosphate binding motif; and LRR, for leucine rich repeats. The ATP binding domain is a small motif of 8 amino acids and starts at position 178. Mutations found in patients with two defective alleles are in red and include nonsense, frameshift, invariant splice site, and missense mutations. Variants found in patients in a heterozygous state and not in controls are mostly missenses and are in blue. Variants found in patients and in subjects from the general population are in black. Mutation nomenclature is according to the Human Genome Variation Society (HGVS) guidelines (http://www.hgvs.org/mutnomen/recs.html).

One difficulty associated with homozygosity mapping and studying rare families is in narrowing down the size of the candidate intervals. This was the case of 19q13.4 candidate region, which was originally four megabases and is a gene-dense region. Consequently, the identification of the causative gene, *NLRP7*, was tedious and required the screening of 80 different genes until the first causative mutations were identified (70). The mutations segregated in the studied families and each patient had two defective alleles, each inherited from one parent as expected for an autosomal recessive disease. Later, others and we confirmed the causality of *NLRP7* mutations in patients from different populations (54, 60, 63, 66, 67, 69, 71, 72), demonstrating that *NLRP7* is a major gene for RHMs. To date, approximately 42 different mutations have been reported in patients with two defective alleles (Figure 3) (73) Of these mutations, 65% are protein-truncating (stop codon, splice mutations, small insertions and deletions, and large rearrangements) and 35% are missense mutations, which are, respectively, higher and lower than the frequencies of these two categories of mutations observed in all human diseases, 56 and 44% (http://www.hgmd.cf.ac.uk/ac/index.php). Although, this difference is not statistically significant, it indicates that patients with RHMs and two mutations may represent the most severe phenotype of the disease.

The identification of *NLRP7* is therefore one of many examples where rare families segregating severe monogenic Mendelian forms of common conditions have led to the identification of causative genes [for an interesting review on the subject see (74)]. This raises an important question: do familial RHM cases with *NLRP7* mutations have more severe mutations than singleton cases? The originally reported families tended to have more protein-truncating mutations than singleton cases. However, this is no longer the case since reports of singleton cases with protein-truncating mutations have increased lately. This could be due to the fact that many singleton cases do not manifest as familial cases because of the small size of families in current societies and/or the lack of other female siblings who have tried to conceive. These factors may have prevented the familial manifestation of many singleton cases with inherited mutations from the two parents.

## *NLRP7* Expression

Before the identification of the causal link between *NLRP7* and RHMs, *NLRP7* transcripts were shown to be expressed in a large number of human tissues including liver, lung, placenta, spleen, thymus, peripheral blood leukocytes, testis, and ovaries (75, 76). After our group linked *NLRP7* to RHMs, we investigated its expression in oocytes and detected its transcripts in all stages of immature oocytes, fertilized eggs, and early embryo cleavage stages (70). These data were later confirmed in an interesting study that showed that *NLRP7* transcripts decrease progressively during oocyte maturation and reach their lowest level on day 3 post-fertilization, which corresponds to the morula stage, then increase sharply from day 3 to day 5, which corresponds to the blastocyst stage and the activation of the fetal genome transcription (77).

At the protein level, *NLRP7* expression was shown in all stages of growing follicles and in all these stages, its expression was restricted to oocytes (72). In another study reported by our group, we detected variable levels of *NLRP7* protein in seven analyzed hematopoietic cells: Epstein Barr virus transformed B-lymphocytes, BJAB, Raji and Ramos (all of B-cell origin), Jurkat (of T-cell origin), and THP1 and U937 (both of monocytic origin) (78).

## *NLRP7* Domains

The *NLRP7* protein consists of three domains: (i) an N-terminal pyrin, (ii) a NACHT termed after four proteins containing an NTPase domain with significant similarities, neuronal apoptosis inhibitor protein (NAIP), MHC class II transcription activators (CIITA), incompatibility locus protein from Podospora anserine (HET-E), and mammalian telomerase-associated proteins (TP1); and (iii) a C-terminal stretch of 9 or 10 leucine rich repeats (LRRs) depending on splice isoforms (Figure 3).

The pyrin domain is a small domain (92 amino acids) found in all *NLRPs* and apoptotic proteins. The pyrin domain functions as an adaptor that helps to connect proteins of the programed death machinery. Pyrin domains can self-associate to form homodimers or associate with other proteins containing structurally related domains to form heterodimers. Domains known to interact with the pyrin domain include the death domain (DD), the death-effector domain (DED), and the caspase activation and recruitment domain (CARD). These pyrin-mediated associations result in the formation of protein complexes and networks that transmit signals from receptors to downstream effectors that function in various cell-death pathways (79). The NACHT domain has an ATP/GTPase-specific P-loop domain, which is a very ancient domain found in bacteria, plants, and all eukaryotes. NTPase domains are found in both apoptotic and anti-apoptotic proteins; they control programed cell-death during development by regulating cytochrome c efflux from the mitochondria, which stimulates apoptosis (80). The LRR domain is found in other proteins with divergent functions such as Toll-like receptors (TLRs), Ran GTPase, and RNAse inhibitor proteins. TLRs are components of the innate immune system, from which the LRR extends into the extracellular milieu where it senses extracellular danger signals and transmits the signals to cytoplasmic proteins. Ran GTPases are essential for transporting RNAs and proteins through the nuclear pore complex by interacting with shuttling transport proteins and changing their ability to bind or release cargo molecules. Finally, RNase inhibitor proteins bind RNAse A and angiogenin and regulate RNA degradation and angiogenesis (81).

## Known Functions and Roles of *NLRP7*

The most studied functions linked to the different NLRP domains are those involved in the activation of the innate immune system in response to various microbial and chemical products. With respect to *NLRP7*, four studies have addressed its functional roles to date and their results are recapitulated in Table 1. Using transient transfections, two studies showed that *NLRP7* down-regulates the intracellular level of mature IL1B (76, 78). While the first study showed that this is due to the down-regulation of pro-IL1B processing (76), the second, by our group, showed that this is due to the lower production of intracellular pro-IL1B (78). In addition, we found that in transient transfections, *NLRP7* inhibition of pro-IL1B production is mediated concomitantly by its three domains, with the strongest effect being mediated by the LRR, followed by the NACHT and the pyrin domains (78). In the study by Kinoshita et al., the authors showed that *NLRP7* binds pro-IL1B and pro-caspase 1 and inhibits IL1B secretion induced by caspase 1, ASC, or NLRP1-delLRR. They also showed that both recombinant mouse IL1B and LPS stimulation enhance *NLRP7* transcription, which in turn down-regulates IL1B secretion. They concluded that *NLRP7* is a negative feedback regulator of IL1B and consequently plays an anti-inflammatory role (76).

**Table 1:** **Recapitulation of the functional roles of *NLRP7* in different studies and cellular models**.

(75)	(76)	(78)		(82)
	LPS or rm-IL1B induce *NLRP7* transcription in PBMC and THP1		

	**Transient transfection in HEK293**		***Ex vivo* PBMC**	**Macrophages**

	*NLRP7* inhibits IL1B secretion induced by NLRP1-delLRR, IL1B, caspase 1, and ASC in transient transfection		**Cells with** ***NLRP7*** **mutations have low secreted IL1B**	***NLRP7*** **silencing reduces IL1B secretion in macrophages**
	***NLRP7*** **down-regulates pro-IL1B** and pro-caspase 1 processing **leading to lower intracellular mature IL1B**	***NLRP7*** **down-regulates pro-IL1B** production **leading to lower intracellular mature IL1B**	*NLRP7* mutations increase slightly pro-IL1B production	
	***NLRP7* interacts with** transfected **pro-caspase 1** and pro-IL1B		*NLRP7* and IL1B subcellular localization overlaps	***NLRP7*** **interacts with caspase 1** and ASC in HEK293 cells through the pyrin domain
			Cells with *NLRP7* mutations have low secreted TNF	*NLRP7* Silencing does not affect IL6 or TNF secretion by macrophages
*NLRP7* silencing with siRNA reduces cellular proliferation				*NLRP7* Silencing with siRNA increases intracellular bacterial growth
				*NLRP7* LRR is necessary to sense bacterial acylated lipopeptides

*LPS stands for lipopolysaccharides; PBMC for peripheral blood mononuclear cells; NLRP1-delLRR stand for NLRP1 in which the leucine rich repeat is deleted; siRNA for small interfering RNA; rm-IL1B, indicates recombinant mouse IL1B. Conclusions obtained by at least two independent studies are in bold character*.

**Figure 4:**
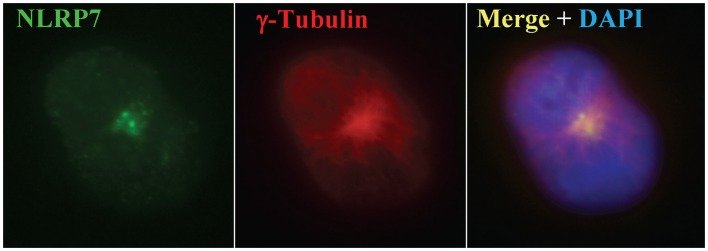
***NLRP7* expression in monocytes using immunofluorescence**. *NLRP7* stains two small dots specific for the microtubule organizing center, which is also revealed with γ-tubulin as previously reported (78).

Part of the study conducted by our group was performed on *ex vivo* LPS-stimulated peripheral mononuclear blood cells from patients with one or two mutations in *NLRP7*. This experiment demonstrated the requirement of wild type *NLRP7* for normal IL1B secretion (78). Within monocytes, which are the main cells that secrete IL1B, *NLRP7* co-localized with the Golgi apparatus and microtubule organizing center (MTOC) (Figure 4) (78). Moreover, treatment of EBV lymphoblastoid cell lines with nocodazole, a drug that depolymerizes microtubules resulted in the fragmentation of *NLRP7* signal. This suggested that normal *NLRP7* associates with microtubules and that its mutations may impair cytokine secretion by disrupting microtubules structures and consequently affecting intracellular trafficking of IL1B vesicles. The role of *NLRP7* in IL1B secretion was confirmed in another independent study involving silencing *NLRP7* in macrophages using small interfering RNA (82). In this study, the effect of silencing eight other *NLRP*s was also tested, but only *NLRP7* knockdown significantly decreased IL1B secretion. This study by Khare et al. also confirmed the physical interaction between *NLRP7*, ASC, and caspase 1 via the pyrin domain, and that the LRR of *NLRP7* is required for sensing bacterial acylated lipopeptides.

Khare et al. (82) also revealed another function of *NLRP7* by demonstrating that *NLRP7* silencing promotes intracellular growth of *Staphylococcus aureus* and *Listeria monocytogenes*. A prior study implying a role for *NLRP7* in cellular proliferation, but in the opposite direction, was reported by Okada et al. (75), who showed that silencing *NLRP7* reduces the proliferation of human embryonal carcinoma cell lines, suggesting that the normal protein promotes cellular growth and has an oncogenic role. The mechanisms leading to both functions are currently unclear and need to be explored in future studies. However, from the HM perspective, we tend to believe in the role suggested by Khare et al. (82), because an important feature of molar tissues from patients with two *NLRP7* defective alleles, which are diploid biparental and obligate carriers of one mutated copy of *NLRP7*, is the excessive proliferation of their trophoblastic cells. This is in line with the data by Khare et al., and is a further indication that *NLRP7* mutations promote cellular growth.

## Understanding the Variability of a Phenotype: Back to the Genetic Complexity of Reproductive Loss

**Figure 5:**
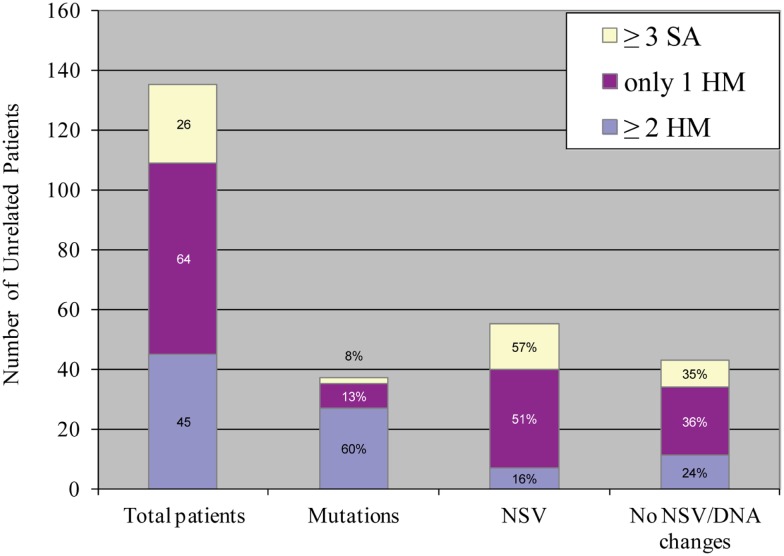
**Summary of *NLRP7* mutation and non-synonymous variants found in 135 unrelated patients with varying histories of reproductive wastage**. HM stands for hydatidiform mole; SA, stands for spontaneous abortion; NSV, for non-synonymous variant. Mutations in *NLRP7* were most frequently observed in patients with at least two HMs, followed by patients with one HM, and then by patients with at least three SAs (84).

**Figure 6:**
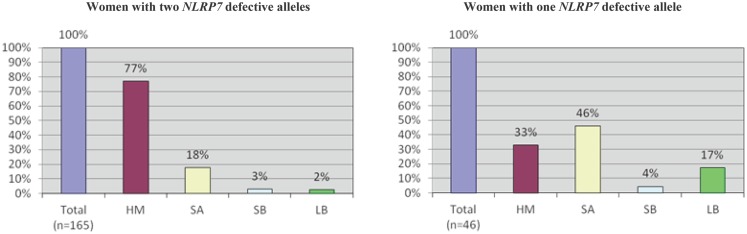
**A comparison of reproductive outcomes between women with two or one defective *NLRP7* allele**. In both histograms, *n* indicates the total number of pregnancies from patients in either category. HM, hydatidiform mole; SA, spontaneous abortion; SB, stillbirth; and LB, live birth. A higher incidence of HMs and a lower incidence of live births are observed in patients with two defective alleles.

An important aspect of our understanding of any disease or system is to understand its variability and determine its extreme phenotypes with its most and less severe manifestations. Despite the fact that we named the 19q13.4 locus as responsible for RHMs, affected patients from the original family, MoLb1, experienced, in addition to their moles, other forms of reproductive loss, namely SAs, stillbirths, an early neonatal death, one malformed live birth, and two live births that led to healthy adults. This large variability in the reproductive outcomes of three patients from MoLb1 was intriguing because such variability is unusual in recessive diseases. However, this variability was not restricted to one family, but was observed, to a lesser extent, in other families studied by our group. Furthermore, this variability was in agreement with data from a large epidemiological study showing increased frequencies of moles, preterm births, stillbirths, and ectopic pregnancies in women with at least two SAs (83). These observations led us to extend our inclusion criteria for *NLRP7* sequencing to women with at least three SAs and no moles as well as to women with the sporadic, common, non-recurrent moles. This analysis showed that two of the 26 analyzed women with recurrent SAs (8%) and eight of the 64 analyzed women with a single HM (associated with and without other forms of reproductive losses) (13%) have novel *NLRP7* non-synonymous variants (NSVs), all missenses in heterozygous state, which were not found in a large number of control subjects from the same ethnicity of the patients (Figure 5) (84). One of the two patients with>3SAs and a missense mutation had a persistent gestational trophoblastic disease requiring chemotherapy after one of her miscarriages. Moreover, six of the patients with one HM and a NSV in *NLRP7* had at least two other reproductive losses, in addition to their HMs, indicating their genetic susceptibility to recurrent reproductive loss. In addition, patients with one defective allele statistically had less severe reproductive outcomes and more live births than patients with two defective alleles (*p*-value = 2.809e−06) (Figure 6).

In conclusion, this analysis did provide a positive answer to our search for mutations in milder phenotype of RHMs. However, it raised challenging questions that all scientists working on complex traits are currently facing: how do we define a pathological NSV? And what tells us that these rare NSVs, found in heterozygous states in a so far believed autosomal recessive disease, have functional consequences on the protein and confer genetic susceptibility for reproductive loss?

## Significance of Rare *NLRP7* NSVs Found in Heterozygous State in Patients

To date, a total of 17 rare NSVs, 16 missenses, and one nonsense, have been observed in heterozygous state in a total of 24 patients but not in controls (67, 85–89) (Figure 3). Some of these NSVs were later found in the 1000 Genomes database but at very low frequencies. Among patients analyzed in our laboratory, 19% of singleton cases with RHMs have one rare NSV in a heterozygous state. At this point in time, it is not clear whether these NSVs are pathologic or not. Consequently, such novel NSVs are not for clinical use and should not be reported to patients to predict the outcomes of future pregnancies. However, they cannot be ignored by scientists aiming at understanding the pathology of RHMs and its relationship to the sporadic common form of HMs, recurrent SAs, and other forms of reproductive loss.

To better understand the significance of these NSVs and elaborate strategies to investigate their pathogenicity, it is important to look at similar situations in other diseases with both rare severe recessive forms and common milder forms. A selection of such diseases is shown in Table 2. The best example is Parkinson disease (PD), for which several causative genes have been identified. Some of these genes are responsible for recessive forms of PD, while others are responsible for dominant forms. Among the causative genes for recessive forms, *PINK1* is responsible for an early onset form of PD and has two mutated alleles in several patients from familial and non-familial sporadic cases of PD. However, other patients were found to have single rare NSVs in heterozygous state. When compared to controls from the same ethnic group, patients with PD were found to have an excess of rare *PINK1* NSVs in heterozygous state. Consequently, these rare NSVs are believed to underlie the genetic susceptibility of these patients for PD (90–92). The same principle applies to other genes: *ATP13A2* responsible for a juvenile onset of PD (93), *GBA* responsible for Gaucher’s disease (94, 95), *ABCA1* responsible for Tangier disease (96), and *MEFV* responsible for familial Mediterranean fever (FMF) (97). In most of these cases, patients with single heterozygous variants have a milder form of the same disease in terms of clinical severity or/and age of onset or have a related condition that include some of the features of the severe disease (93–97).

**Table 2:** **Examples of genes causing rare severe recessive diseases and confering susceptibility to common or related forms of the same disease**.

Gene	Two defective alleles	Single mutated allele	Reference
*PINK1*	Autosomal recessive Parkinson diease (PD) with early onset	More rare variants in patients vs. controls (10 vs. 2)	(90, 92)
		Milder phenotype and later onset in heterozygous relatives of severely affected patients in large pedigrees	(91)
*ATP13A2*	Juvenile onset Parkinson disease<21 years	Young onset Parkinson disease	(93 )
*GBA*	Gaucher’s disease	More rare variants in patients with PD vs. controls. This seems specific to some ethnic groups, e.g., Ashkenas, French	(94, 95)
*MEFV*	Familial mediterranean fever	In 15% of patients	(97)
*ABCA1*	Familial hypoalphalipoproteinemia	More rare variants in individuals with low HDL-C than in those with high HDL-C (16% vs. 2%)	(96)

With respect to RHMs, the age of onset is not an appropriate indicator of severity; however, a severe genetic defect would translate into recurrence and would be expected to lead to the same genetic defect every time a patient tries to conceive. On the contrary, a milder genetic defect, which can be modulated by other environmental factors, would be expected to lead to more variability in the reproductive outcomes of the patients. This is exactly the conclusion we reached in the last analysis performed on three categories of patients (RHM, sporadic HM, and recurrent SA), which showed that patients with RHM have the highest frequency of *NLRP7* mutations (60%), and these patients had mostly two defective alleles, each. However, 13% of patients with one mole and other reproductive wastage had a single variant in a heterozygous state, while 8% of patients with at least three SAs had rare *NLRP7* variants in heterozygous state (Figure 5). Similar results were obtained from patients with sporadic HM and reproductive wastage in a different population (Tunisian) and again showed the presence of *NLRP7* variants in heterozygous state in 13% of the patients (59). Additional case-control studies designed to screen all *NLRP7* exons in patients with sporadic HM and recurrent SAs are needed to assess whether the burden of *NLRP7* mutations and rare NSVs is higher in patients than in ethnically matched controls. In the meantime, a number of other tests can be used to investigate the pathogenicity of encountered variants. These include (i) the absence of the variants in controls of matching ethnicity to the patients; (ii) the conservation of the changed amino acids throughout evolution; (iii) the predicted functional consequences of the identified variants using various algorithm; (iv) the segregation of the variants on different haplotypes when present with other known deleterious mutations; (v) the functional impact of the variants on the protein subcellular localization; and ideally (vi) the impact of the variants on the protein function in any type of cellular assays.

## Genotype of HM Tissues in Patients with *NLRP7* Mutations

To date, the parental contribution to approximately 70 HM tissues from patients with two defective alleles in *NLRP7* have been characterized and all of them were found to be diploid biparental (55, 62, 63, 87, 98–100) with the exception of one tissue that was digynic (101). However, this is not the case for HM tissues from patients with single heterozygous rare *NLRP7* variants. In this category of patients, few HM tissues were genotyped; some were found to be diploid androgenetic monospermic (67, 85, 87, 89) and others were found to be triploid diandric dispermic (102). The reason for this difference is not yet clear and needs to be addressed in future studies. Such studies may also clarify whether specific single heterozygous rare *NLRP7* variants confer a genetic susceptibility to a specific genotypic type of moles. This would help elucidating the mechanisms of the formation of different genotypic types of moles. This is particularly important because the currently accepted mechanisms of mole formation are hypothetical and the emerging ideas propose a single model stemming from dispermic fertilization followed by postzygotic abnormalities (33).

## *NLRPs* and Reproduction

### Nlrp5

*Nlrp5* (originally called *Op1* then *Mater*, and lately *Nlrp5*) is the first *NLRP* gene shown to play a causative role in mammalian reproduction (103). *Nlrp5* was isolated from a mouse model of autoimmune oophoritis (also termed premature ovarian failure) generated by neonatal thymectomy. Female mice thymectomized in the third day after birth spontaneously develop autoimmune disorders characterized by organ-specific inflammation and lymphocyte infiltration (104). In some mouse strains, the predominant autoantibody is directed against the ovary where it reacts with NLRP5. To gain insights about the role of *NLRP5* in autoimmune oophoritis, the authors generated knockout null females, *NLRP5*^−/−^, and found that these females ovulate normally and their oocytes fertilize *in vivo* with no apparent abnormalities. However, their embryos stop developing at the two-cell stage, a time at which major embryonic genome activation takes place. The role of *NLRP5* in preimplantation embryonic development was also confirmed in monkeys where its knockdown in MII oocytes resulted in a significant reduction in the number of embryos that reached the blastocyst stage (105). In mouse oocytes, *NLRP5* is part of specialized oocyte cytoskeletal structures (called cytoplasmic lattices) that are responsible for the distribution of organelles, maternal mRNA, and maternal proteins in the oocytes (106–108). Also, previous studies on *NLRP5* showed that within oocytes, *NLRP5* localizes to mitochondria and nuclear pores and is implicated in oxidative stress during oocyte aging (109).

### NLRP14

To date, a single study has implicated *NLRP14* in spermatogenic failure in humans based on the presence of one stop codon and four missense mutations, all of which were found in heterozygous state and each in a single patient and were not found in controls (110). However, no additional studies replicating the causal role of *NLRP14* or explaining its potential role in spermatogenic failure have been reported.

### NLRP2

*NLRP2* is the closest human gene to *NLRP7* in terms of protein homology and both genes are believed to have originated from the same mouse paralog during evolution (109, 111–113). *NLRP2* was shown to be responsible for a single familial case of Beckwith–Wiedemann syndrome (BWS) based on the presence of a frameshift mutation in a homozygous state in an unaffected mother and in her two children affected with BWS (114). The presence of a homozygous *NLRP2* mutation in the mother of two children with BWS is interesting because of the relationship between BWS and HM, and their association with reproductive loss and abnormal imprinting. However, since that report, no other cases of BWS were shown to have mutations in *NLRP2*, which makes this finding either a rare causal event occurring in a small minority of cases or a coincidental association. In addition, *Nlrp2* knockdown in murine oocytes at the germinal vesicle stage was shown to lead to embryonic arrest at the two-cell stage (115).

### Nlrp4e

Recently a new study investigating the role of mouse *Nlrp4e* in female reproduction has been reported. In this study, *Nlrp4e* was found expressed in all follicular stages, unfertilized eggs, and early embryo cleavage stages. Again, *Nlrp4e* knockdown in fertilized eggs resulted in a reduced number of embryos that reach the blastocyst stage, which is an indication that maternal *Nlrp4e* is required for early embryo development (116).

## Conclusion

Since the identification of *Nlrp5* and *NLRP7*, the list of *NLRP* genes with maternal-effects continues to grow. We expect this list to expand even further because of the presence of four additional *NLRPs* besides *NLRP4* and *NLRP2* that show oocyte-specific expression and have not yet been linked to reproduction in any organism: *NLRP8*, 9, 11, and 13 (112). All of these *NLRP*s are highly expressed in germinal vesicle oocytes and decrease during preimplantation development to reach their lowest levels at the blastocyst stage, which is in favor of their maternal-effect role.

With respect to *NLRP7*, we do not yet know the exact role of its protein in human oocytes. However, based on several observations, we believe that oocytes from patients with mutations are defective at several levels and are not able to sustain early embryonic development. Consequently, the embryos stop developing very early in these conceptions. Because these patients also have decreased cytokine secretion, we believe that they fail to mount an appropriate inflammatory response to reject these arrested pregnancies as normal women would. As a result, the retention of these dead pregnancies with no embryos to later gestational stages leads to the hydropic degeneration of CV. This, combined with the potential role of *NLRP7* mutations in enhancing proliferation, may lead to the three fundamental aspects of moles: aberrant human pregnancies with no embryo, abnormal excessive trophoblastic proliferation, and hydropic degeneration of CV. We believe that fully understanding the three aspects of the pathology of HM would greatly benefit from collaborations between scientists in various medical fields.

## Conflict of Interest Statement

The authors declare that the research was conducted in the absence of any commercial or financial relationships that could be construed as a potential conflict of interest.
